# Multidimensional Approach to Exploring Neighborhood Determinants and Symptom Severity Among Individuals With Psychosis

**DOI:** 10.1001/jamanetworkopen.2024.10269

**Published:** 2024-05-15

**Authors:** Oladunni Oluwoye, Megan Puzia, Ari Lissau, Ofer Amram, Douglas L. Weeks

**Affiliations:** 1 Department of Community and Behavioral Health, Elson S. Floyd College of Medicine, Washington State University, Spokane; 2 Department of Nutrition and Exercise Physiology, Elson S. Floyd College of Medicine, Washington State University, Spokane; 3 Paul G. Allen School for Global Animal Health, Washington State University, Pullman

## Abstract

**Importance:**

The impact of cumulative exposure to neighborhood factors on psychosis, depression, and anxiety symptom severity prior to specialized services for psychosis is unknown.

**Objective:**

To identify latent neighborhood profiles based on unique combinations of social, economic, and environmental factors, and validate profiles by examining differences in symptom severity among individuals with first episode psychosis (FEP).

**Design, Setting, and Participants:**

This cohort study used neighborhood demographic data and health outcome data for US individuals with FEP receiving services between January 2017 and August 2022. Eligible participants were between ages 14 and 40 years and enrolled in a state-level coordinated specialty care network. A 2-step approach was used to characterize neighborhood profiles using census-tract data and link profiles to mental health outcomes. Data were analyzed March 2023 through October 2023.

**Exposures:**

Economic and social determinants of health; housing conditions; land use; urbanization; walkability; access to transportation, outdoor space, groceries, and health care; health outcomes; and environmental exposure.

**Main Outcomes and Measures:**

Outcomes were Community Assessment of Psychic Experiences 15-item, Patient Health Questionnaire 9-item, and Generalized Anxiety Disorder 7-item scale.

**Results:**

The total sample included 225 individuals aged 14 to 36 years (mean [SD] age, 20.7 [4.0] years; 152 men [69.1%]; 9 American Indian or Alaska Native [4.2%], 13 Asian or Pacific Islander [6.0%], 19 Black [8.9%], 118 White [55.1%]; 55 Hispanic ethnicity [26.2%]). Of the 3 distinct profiles identified, nearly half of participants (112 residents [49.8%]) lived in urban high-risk neighborhoods, 56 (24.9%) in urban low-risk neighborhoods, and 57 (25.3%) in rural neighborhoods. After controlling for individual characteristics, compared with individuals residing in rural neighborhoods, individuals residing in urban high-risk (mean estimate [SE], 0.17 [0.07]; *P* = .01) and urban low-risk neighborhoods (mean estimate [SE], 0.25 [0.12]; *P* = .04) presented with more severe psychotic symptoms. Individuals in urban high-risk neighborhoods reported more severe depression (mean estimate [SE], 1.97 [0.79]; *P* = .01) and anxiety (mean estimate [SE], 1.12 [0.53]; *P* = .04) than those in rural neighborhoods.

**Conclusions and Relevance:**

This study found that in a cohort of individuals with FEP, baseline psychosis, depression, and anxiety symptom severity differed by distinct multidimensional neighborhood profiles that were associated with where individuals reside. Exploring the cumulative effect of neighborhood factors improves our understanding of social, economic, and environmental impacts on symptoms and psychosis risk which could potentially impact treatment outcomes.

## Key Points

**Question:**

Is the cumulative exposure to neighborhood-level socioeconomic and environmental factors among individuals with first-episode psychosis associated with psychosis, depression, and anxiety symptom severity at intake?

**Findings:**

In this cohort study with a sample of 225 participants with first-episode psychosis, with approximately half of individuals residing in urban high-risk neighborhoods, residents of urban high-risk neighborhoods had more severe symptom severity at intake than individuals residing in rural neighborhoods.

**Meaning:**

These results suggest that the combination of various intersecting neighborhood factors are associated with symptom severity prior to the initiation of coordinated specialty care.

## Teaser

This cohort study of US adolescents and adults who experienced first-episode psychosis examines associations of neighborhood-level socioeconomic and environmental factors with symptom severity.

## Introduction

Contextual factors, such as neighborhood-level factors, cover a broad range of societal, socioeconomic, and political conditions, including urbanicity, housing instability, pollution, social fragmentation, and access to resources and services.^1^ The neighborhoods in which people reside present exposure to multiple and intersecting factors associated with mental health.^2,3^ For example, residing in certain neighborhoods can lead to aggravation of risk factors for mental health conditions and increase the severity of mental health conditions, including those associated with early psychosis.^4,5,6^ Focusing on how one’s physical environment (ie, neighborhood) affects psychosis and mental health provides an alternative lens that moves beyond our understanding of how individual-level factors alone are associated with severity of symptoms and psychosis risk.

Studies have demonstrated the relationship between urbanicity, exposure to air pollutants,^7,8^ socioeconomic area deprivation,^9^ neighborhood crime,^10^ inequality,^11^ social fragmentation,^12^ and psychotic experiences and symptom severity. Regarding urbanicity, findings from a meta-analysis reported a higher risk for schizophrenia among individuals residing in more urban communities relative to those residing in rural areas.^13^ A 2021 study^14^ found air pollutant levels of particulate matter—including nitrogen dioxide, oxides, and ozone—was associated with increased odds of psychosis. Findings have also found the exposure to air pollution is associated with higher odds of depression^15^ and anxiety symptom severity.^16^ While urbanicity has been found to be related to psychotic experiences, research has demonstrated the relationship between urbanicity and psychotic experiences may be explained by other factors such as exposure to air pollutants and social fragmentation.^17,18^ Studies to date have primarily explored singular neighborhood factors and not multidimensional factors when examining their relationship to symptom severity and psychosis risk. However, it is plausible that there is high correlation between multiple neighborhood factors and that previous research has not captured the cumulative exposure of multiple factors on symptom severity.^19^ This is important to address as multiple factors are more representative of where people reside, thus providing a more accurate assessment of neighborhood risk.

The current study sought to examine the association between multidimensional neighborhood characteristics and depression, anxiety, and psychosis symptom severity at intake in a cohort of individuals with first-episode psychosis (FEP). The study aimed to (1) identify latent profiles of neighborhood clusters based on unique combinations of shared social, economic, and environmental neighborhood factors, and (2) validate these profiles by identifying differences in mental health symptom severity.

## Methods

### Sampling and Setting

This cohort study used data collected from the New Journeys network, Washington State’s coordinated specialty care (CSC) model implemented at 14 sites. The New Journeys model uses person-centered approaches to deliver evidence-based psychosocial interventions and low-dose antipsychotic medication to individuals aged 15 to 40 years with nonaffective psychosis for less than 2 years.^20^ Individuals with substance-induced psychosis, mood disorder with psychotic features, diagnosed autism spectrum disorder, or pervasive developmental disorder were not eligible for New Journeys. Individuals complete a standardized assessment battery at enrollment (ie, baseline) that gathers sociodemographic characteristics and several validated and reliable self-administered questionnaires on recent mental health experiences.^19^ From January 2017 to August 2022, mental health symptom severity at intake was collected from 225 individuals enrolled in New Journeys.

The Strengthening the Reporting of Observational Studies in Epidemiology (STROBE) reporting guideline for cohort studies was used. The data used in the present study was collected as part of the New Journeys network evaluation, which obtained an exemption of consent in the use of deidentified data from the Washington State institutional review board.

### Measures

#### Sociodemographic Characteristics

Ethnic and racial identity was collected via self-report and categories included: American Indian or Alaska Native, Asian, Black, Pacific Islander, White, multiracial, and other. Ethnicity was categorized as Hispanic (or Latinx) or non-Hispanic. Additional characteristics included age, gender (men, women, and nonbinary or other), educational level, household income, and health insurance status. Residential addresses were geocoded using R and mapped in ArcGIS to determine the census tract of the residence.^21,22^

#### Baseline Mental Health Measures

Depression and anxiety were assessed using the 9-item Patient Health Questionnaire (PHQ-9), in which scores range from 0 to 27, with higher scores corresponding with criteria in the *Diagnostic and Statistical Manual of Mental Disorders* (Fourth Edition) for major depressive disorder^23^; and the 7-item Generalized Anxiety Disorder (GAD-7), in which scores range from 0 to 21, with higher scores indicating greater severity of symptoms.^24^ The 15-item Community Assessment of Psychic Experiences-Positive scale (CAPE-P15) was administered to assess recent symptoms of psychosis with scores ranging from 1 to 4.^25,26^

#### Neighborhood Indicators

Guided by prior research, 12 indicators were selected to characterize neighborhood-level factors across key domains known to influence mental health outcomes: rurality, outdoor recreational space, walkability, grocery access, diversity of land use, transportation barriers, health care shortages, housing conditions, health outcomes, economic and social determinants of health, and environmental exposures.^6,8,27^ For each indicator, the raw data values were converted to decile rankings for each census tract. Data for most of our variables—areas with health care shortages, access to outdoor recreational space, land-use mix, housing conditions, health outcomes, environmental exposure, economic determinants, and social determinants—were accessed via the Washington Tracking Network (WTN), a state-level system comprising a collection of data sets related to public health. Data within the WTN are compiled from a variety of sources, including environmental monitoring stations, geographic information system locators, hospital admissions, and the US census and American Community Survey. Health care shortage areas were determined based on the number of clinicians (ie, primary health care, mental health, and dental care) per capita and information on the intensity of the need for clinicians in that area. Land use mix was calculated as a component of a walkability score consistent with previous research.^28^ Raw scores reflect the diversity of land uses within a tract and range from zero (being only 1 land use type) to 100 (equal distribution of 6 land use types: education, entertainment, single-family residence, multi-family residence, retail, office). Access to outdoor recreation space was determined by creating a 1-mile buffer around all parks and calculating the percentage area of each census block that is within a park buffer. The corresponding percentage of the population younger than 18 years old within a park buffer was calculated by taking the total population younger than age 18 years divided by the total population for all census blocks that make up a census tract (eMethods in Supplement 1). In addition to single-variable indicators and raw data, the WTN includes composite variables of aggregated indicators (accessible via their Information by Location mapping tool). As means of efficiently characterizing multidimensional concepts, composite variables were used to describe economic determinants (eg, children living in poverty, single-parent households), social determinants (eg, limited English proficiency, no high school diploma), health outcomes (eg, life expectancy at birth, premature death), housing conditions (eg, housing lead risk, unaffordable housing), and environmental exposure (eg, ozone concentration, proximity to heavy traffic roadways) (eTables 1 through 5 in Supplement 1). Composite variables aggregately measure related facets with minimal overlap of specific contributors within and across variables (eMethods in Supplement 1).

The Rural-Urban Commuting Area (RUCA) taxonomy developed by the US Department of Agriculture (USDA) categorizes US census tracts based on the degree of urbanization and commuting patterns.^29^ Categorically, codes are grouped between 1 and 3 (metropolitan areas), between 4 and 6 (micropolitan areas), between 7 and 9 (small-town areas), and 10 (rural areas). Grocery store access was measured as the percentage of the population living farther than 1 mile away from a supermarket using data from the Food Access Research Atlas developed by the USDA.^30^ Walkability scores were obtained from the US Environmental Protection Agency’s National Walkability Index and based on factors like residential density, street connectivity, land-use mix, and retail area.^31^ Transportation barrier scores were captured using the Washington State Department of Transportation’s census tract scores, which quantify the difficulty of travel to essential services based on road metrics, transit availability, and travel time to key destinations like grocery stores, doctors, and schools.^32^ eMethods in Supplement 1 provides additional details on metrics and original sources.

### Statistical Analyses

Latent profile analysis (LPA) was conducted using the TidyLPA package in R version 4.2 (R Project for Statistical Computing).^33,34^ The sample consisted of the unique 1458 census tracts in Washington state, with no missing data. Models were estimated using maximum likelihood, with variances constrained to be equal across classes with covariances fixed to zero. Multiple fit statistics were considered when determining the number of profiles, including Akaike information criterion (AIC), approximate weight of evidence criterion (AWE), bayesian information criterion (BIC), classification likelihood criterion (CLC), Kullback information criterion (KIC), and bootstrapped likelihood ratio tests (BLRTs). Solutions with 2 to 8 profiles were investigated. To avoid local solutions, the model was run with multiple random starting values. Posterior probabilities were used to assign census tracts to their most likely profile membership.

The statewide neighborhood profiles identified through LPA were validated by assessing differences in depression, anxiety, and psychosis scores among participants residing in each profile. Profile validation analyses were conducted in SPSS version 29.0 (IBM Corp). To account for the nested data structure and treatment site effects, general linear mixed models (GLMMs) were used to evaluate the associations of LPA with baseline depression, anxiety, and psychosis symptom severity scores. Census tract neighborhood profile membership, based on geocoded residential addresses, was included as a fixed-effect factor of interest. The neighborhood profile with the lowest risk served as the reference group. Age, gender, race, ethnicity, education level, and household income were included as fixed-effect covariates, and site as a random effect. Models were estimated using maximum likelihood and robust standard errors. Tests were 2-sided, and a *P* < .05 was considered statistically significant. Corrected *P* values for the neighborhood type variable were obtained using the Benjamini-Hochberg procedure to account for multiple testing across positively correlated outcomes.^35^

## Results

### Descriptive Statistics

**Figure 1.**
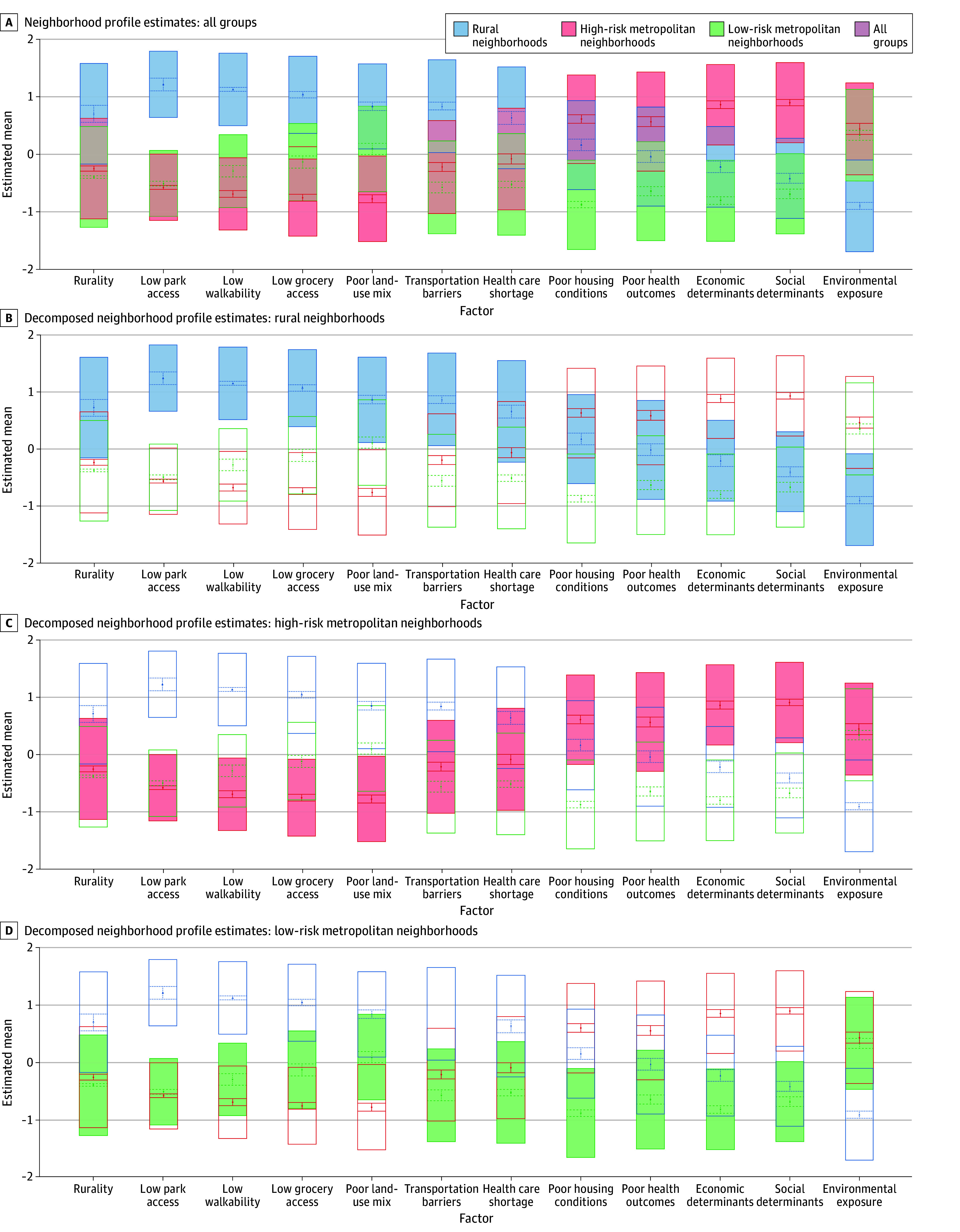
Neighborhood Typologies and Profile Estimates for 3-Class Solution See the Methods section and eMethods in Supplement 1 for additional details on metrics and original sources.

The study sample included 225 individuals with FEP (152 men [69.1%]; 9 American Indian or Alaska Native [4.2%], 13 Asian or Pacific Islander [6.0%], 19 Black [8.9%], and 118 White [55.1%]; 55 Hispanic ethnicity [26.2%]) with the mean (SD) age of 20.7 (4.0) years (Table 1). In LPAs, all 1458 state-level census tracts had sufficient data for analysis. We investigated the fit statistics for solutions with 2 to 8 profiles, at which point the addition of further classes did not produce significant BLRTs (eTable 2 and the eFigure in Supplement 1). Overall, a 3-class solution was determined to be the most appropriate for classifying neighborhood typologies, labeled as urban low-risk, urban high-risk, and rural. Although model fit statistics were somewhat improved with the addition of a fourth class (and subsequent classes), interpretability of a fourth profile was limited, as it largely functioned to separate the high- and low-risk urban areas into profiles that may be broadly described as high-, low-, and medium-risk, but were less distinct than the groups observed in the 3-class solution. Profiles for the 3-class solution are depicted in Figure 1.

**Table 1.** Sociodemographic Characteristics

Characteristic	Individuals, No. (%) (N = 225)^a^
Gender	
Men	152 (69.1)
Women	60 (27.3)
Nonbinary or other gender	8 (3.7)
Race	
American Indian or Alaska Native	9 (4.2)
Asian or Pacific Islander	13 (6.0)
Black	19 (8.9)
White	118 (55.1)
Other	39 (18.2)
Multiracial	16 (7.5)
Hispanic ethnicity	55 (26.2)
Age, mean (SD), y	20.7 (4.0)
Education	
No high school degree	45 (36.9)
High school	36 (29.5)
Some college	32 (26.2)
College degree	9 (7.3)
Household income	
<$20 000	22 (23.4)
$20 000-$49 999	37 (41.4)
$50 000-$74 999	15 (16.0)
$75 000-$99 999	13 (13.8)
≥$100 000	5 (5.3)
Receiving state and/or federal income support	57 (25.3)
Insurance type	
Private	50 (22.7)
Public	162 (73.6)
No health insurance	8 (3.6)
Housing situation	
Stable housing	192 (86.5)
Institution	15 (6.8)
Temporary or unstable	13 (5.9)
Homeless	2 (0.9)

^a^ Percentages reflect the proportion of the sample that gave valid responses.

Table 2 depicts LPA indicator estimates for neighborhood profiles. Class 1, urban low-risk (532 tracts [36.5%]; 2 687 369 Washington residents [36.3%]), consisted of nonrural areas with relatively high income, high educational attainment, high access to transportation and health care, but relatively high levels of environmental exposure. Class 2, urban high-risk (486 tracts [33.3%]; 2 628 814 Washington residents [35.5%]), consisted of tracts that are nonrural, with relatively low income, low educational attainment, and high access to transportation, but low access to health care and relatively high levels of environmental exposure. Finally, class 3, rural (440 tracts [30.2%]; 2 087 924 of Washington residents [28.2%]), consisted of rural areas and smaller towns with average income, average to low educational attainment, relatively limited access to health care and transportation, but low levels of environmental exposure. Participants resided in 196 census tracts. Approximately a quarter of the sample (56 [24.9%]) resided in urban low-risk neighborhoods, approximately half (112 [49.8%]) in urban high-risk neighborhoods, and approximately a quarter (57 [25.3%]) in rural neighborhoods.

**Table 2.** Means and Variances From LPA of Washington Neighborhoods

Indicator^a^	LPA mean estimates						Variance	
	Urban low-risk (n = 532)		Urban high-risk (n = 486)		Rural (n = 440)		Estimate (SE)	*P* value
	Estimate (SE)	*P* value	Estimate (SE)	*P* value	Estimate (SE)	*P* value		
RUCA	−0.39 (0.01)	<.001	−0.25 (0.03)	<.001	0.71 (0.08)	<.001	0.77 (0.05)	<.001
Limited green space	−0.51 (0.02)	<.001	−0.57 (0.02)	<.001	1.22 (0.06)	<.001	0.33 (0.02)	<.001
Low walkability	−0.29 (0.05)	<.001	−0.69 (0.03)	<.001	1.14 (0.02)	<.001	0.40 (0.02)	<.001
Low grocery access	−0.13 (0.05)	.02	−0.75 (0.03)	<.001	1.04 (0.03)	<.001	0.45 (0.02)	<.001
Poor land-use mix	0.10 (0.05)	.04	−0.77 (0.04)	<.001	0.84 (0.04)	<.001	0.56 (0.02)	<.001
Transportation barriers	−0.57 (0.05)	<.001	−0.21 (0.04)	<.001	0.85 (0.03)	<.001	0.66 (0.03)	<.001
Health care shortage	−0.52 (0.03)	<.001	−0.08 (0.04)	.07	0.64 (0.06)	<.001	0.79 (0.03)	<.001
Poor housing conditions	−0.88 (0.03)	<.001	0.62 (0.04)	<.001	0.16 (0.05)	.002	0.60 (0.02)	<.001
Poor health outcomes	−0.64 (0.04)	<.001	0.57 (0.04)	<.001	−0.03 (0.05)	.51	0.77 (0.05)	<.001
Economic determinants	−0.81 (0.03)	<.001	0.87 (0.04)	<.001	−0.22 (0.05)	<.001	0.33 (0.02)	<.001
Social determinants	−0.68 (0.04)	<.001	0.91 (0.03)	<.001	−0.41 (0.04)	<.001	0.40 (0.02)	<.001
Environmental exposure	0.34 (0.04)	<.001	0.45 (0.05)	<.001	−0.90 (0.03)	<.001	0.45 (0.02)	<.001

Abbreviations: LPA, latent profile analysis; RUCA, Rural-Urban Commuting Area.

^a^ Economic determinants, social determinants, nearby housing conditions, area environmental exposure, and population health outcomes were composite variables extracted via the Washington Tracking Network’s (WTN) Information by Location mapping tool. Limited green space, land-use mix, and health care shortage were accessed from WTN tables. Rurality was determined using Rural-Urban Commuting Area (RUCA) taxonomy. Grocery access was measured from the Food Access Atlas. Walkability scores were obtained from the National Walkability Index. Transportation barriers were quantified using the Department of Transportation’s census tract scores. See eTables 1 through 5 in Supplement 1 for additional details on metrics and original sources. Variances and covariances were constrained to be equal across groups.

### Baseline Psychosis, Depression, and Anxiety Symptom Severity by Neighborhood Profiles

**Figure 2.**
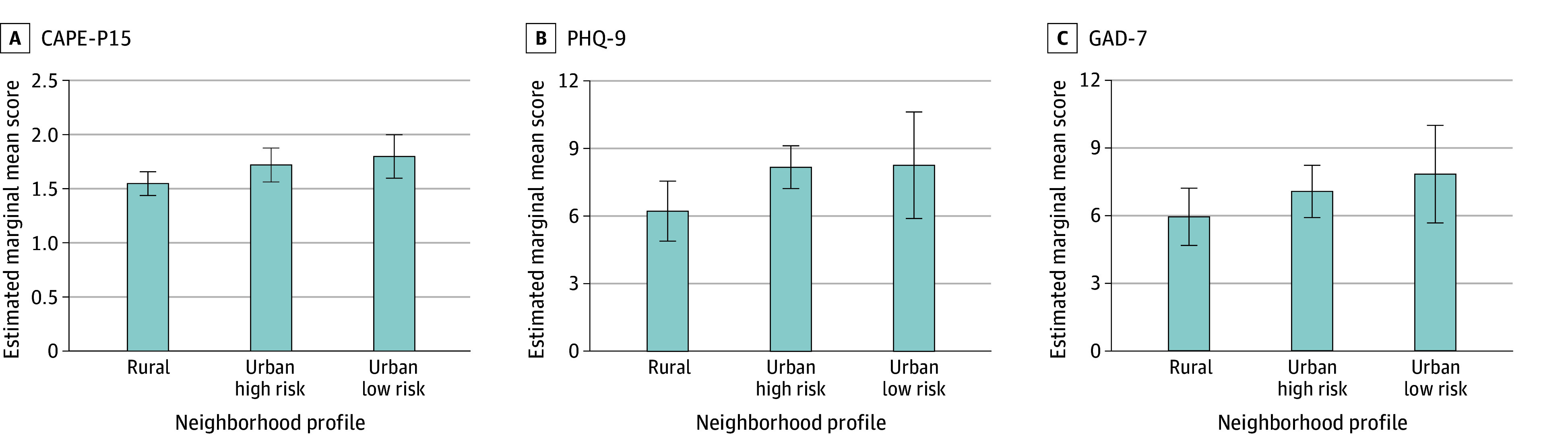
Estimated Marginal Means From Generalized Linear Mixed Models of Participant Mental Health Outcomes All neighborhoods are characterized at the census-tract level, based on tract boundaries from the 2010 US decennial census. CAPEP-15 indicates Community Assessment of Psychotic Experiences-15; GAD-7, Generalized Anxiety Disorder-7; PHQ-9, Patient Health Questionnaire-9.

GLMMs including neighborhood profile and individual-level covariates were associated with mental health symptoms at CSC intake. At the individual level, psychosis symptom severity significantly differed based on gender (higher mean scores among participants who identified as women, nonbinary, or other vs men: estimate [SE], 0.16 [0.07]), race (lower mean scores among those identified as other racial categories [ie, American Indian or Alaska Native, Asian, Black, Pacific Islander, multiracial, or other] vs White: estimate [SE], −0.19 [0.10]), education (higher mean scores among those without a high school degree: estimate [SE], 0.25 [0.07]), and income (lower mean scores among those with higher annual household income: estimate [SE], −0.05 [0.02]) (Table 3). Psychosis symptom severity was not associated with ethnicity or age. While controlling for individual factors, there were significant differences in psychosis symptom severity based on neighborhood profile (Benjamini-Hochberg–corrected *P* = .03). Specifically, participants residing in urban high-risk (estimate [SE], 0.17 [0.07]) and urban low-risk (estimate [SE], 0.25 [0.12]) neighborhoods had higher CAPE-P15 scores than those residing in rural neighborhoods (Figure 2).

**Table 3.** Estimates From Generalized Linear Mixed Models of Participant Mental Health Outcomes

Parameter^a^	Estimate (SE) [95% CI]	β	*P* value
**CAPE-P15**			
Intercept	1.86 (0.19) [1.49 to 2.22]	NA	<.001
Women, nonbinary, or other gender	0.16 (0.07) [0.03 to 0.30]	0.14	.02
American Indian or Alaska Native, Asian, Black, Pacific Islander, multiracial, or other	−0.19 (0.10) [−0.38 to 0.00]	−0.17	.047
Income	−0.05 (0.02) [−0.08 to −0.01]	−0.15	.01
Age	−0.05 (0.04) [−0.13 to 0.03]	−0.10	.20
Hispanic ethnicity	0.21 (0.13) [−0.05 to 0.47]	0.17	.11
No high school degree	0.25 (0.07) [0.11 to 0.39]	0.22	<.001
Urban high-risk profile	0.17 (0.07) [0.04 to 0.30]	0.14	.01
Urban low-risk profile	0.25 (0.12) [0.01 to 0.49]	0.22	.04
**PHQ-9**			
Intercept	5.99 (1.88) [2.30 to 9.68]		.002
Women, nonbinary, or other gender	3.15 (0.63) [1.92 to 4.39]	0.25	<.001
American Indian or Alaska Native, Asian, Black, Pacific Islander, multiracial, or other	−2.19 (1.04) [−4.23 to −0.15]	−0.18	.04
Income	0.21 (0.18) [−0.15 to 0.57]	0.06	.25
Age	−0.23 (0.54) [−1.28 to 0.82]	−0.04	.66
Hispanic ethnicity	0.43 (0.80) [−1.15 to 2.01]	0.03	.59
No high school degree	−0.46 (0.91) [−2.25 to 1.34]	−0.04	.62
Urban high-risk profile	1.97 (0.79) [0.42 to 3.52]	0.15	.01
Urban low-risk profile	2.05 (1.15) [−0.21 to 4.31]	0.17	.08
**GAD-7**			
Intercept	5.34 (2.31) [0.80 to 9.87]	NA	.02
Women, nonbinary, or other gender	2.66 (0.68) [1.32 to 4.00]	0.23	<.001
American Indian or Alaska Native, Asian, Black, Pacific Islander, multiracial, or other	−2.23 (0.82) [−3.85 to −0.62]	−0.20	.01
Income	0.30 (0.15) [0.01 to 0.60]	0.09	.04
Age	0.19 (0.56) [−0.90 to 1.28]	0.04	.73
Hispanic ethnicity	2.20 (1.03) [0.17 to 4.22]	0.17	.03
No high school degree	−0.21 (0.78) [−1.75 to 1.33]	−0.02	.79
Urban high-risk profile	1.12 (0.53) [0.07 to 2.18]	0.09	.04
Urban low-risk profile	1.89 (1.32) [−0.71 to 4.49]	0.17	.15

Abbreviations: CAPE-P15, Community Assessment of Psychotic Experiences-15; GAD-7, Generalized Anxiety Disorder-7; NA, not applicable; PHQ-9, Patient Health Questionnaire-9.

^a^ All neighborhoods are characterized at the census-tract level, based on tract boundaries from the 2010 US decennial census.

Severity of depression symptoms was significantly associated with gender (higher among participants who identified as women, nonbinary or other vs men: estimate [SE], 3.15 [0.63]) and race (lower among those identified as other racial categories vs White: estimate [SE], −2.19 [1.04]), but was not associated with education, income, age, or ethnicity (Table 3). Controlling for individual factors, there were also significant differences in depression symptom severity based on neighborhood profile (Benjamini-Hochberg–corrected *P* = .02). Specifically, participants residing in urban high-risk neighborhoods had more severe depression symptoms than those residing in rural neighborhoods (estimate [SE], 1.97 [0.79]); participants residing in urban low-risk neighborhoods did not significantly differ from those in rural neighborhoods.

Severity of anxiety symptoms significantly differed based on gender (higher scores among participants who identified as women, nonbinary, or other vs men: estimate [SE], 2.66 [0.68]), race (lower scores among those identified as other racial categories vs White: estimate [SE], −2.23 [0.82]), ethnicity (higher among those identified as non-Hispanic vs Hispanic: estimate [SE], 2.20 [1.03]), and income (higher scores among those with lower annual household income: estimate [SE], 0.30 [0.15]), but not age or education (Table 3). Controlling for individual factors, there were also significant differences in anxiety symptom severity based on neighborhood profile (Benjamini-Hochberg–corrected *P* = .04). Specifically, participants living in urban high-risk neighborhoods had more severe anxiety symptoms than those residing in rural neighborhoods; participants residing in urban low-risk neighborhoods did not differ from those in rural neighborhoods.

## Discussion

To our knowledge, this study is the first to empirically identify neighborhood profiles based on unique social, economic, and environmental typologies used to examine the intersecting determinants that are associated with symptom severity in a high risk-clinical sample. Our LPA identified 3 distinct multidimensional neighborhood profiles across Washington state. In our participant sample, we found that approximately 50% of participants resided in urban high-risk neighborhoods (compared with 36% of Washington residents statewide), 25% resided in urban low-risk neighborhoods, and 25% in rural neighborhoods. Our findings revealed important differences in symptom severity prior to initiation of CSC based on where individuals reside. In previous research, exposure to air pollutants, area deprivation, and urbanicity have each independently been associated with psychosis risk and anxiety and depression symptom severity.^8,9,11^ However, results from our study show that operationalizing neighborhoods as a multidimensional concept that incorporates the unique intersections of individual-level determinants may further illuminate findings on psychosis risk from single-indicator studies. We found that compared with rural neighborhoods, neighborhoods characterized by urbanicity and environmental exposure (ie, air and noise pollution) were unilaterally associated with more severe psychotic symptoms, but that more severe depression and anxiety symptoms were specific to individuals residing in urban neighborhoods that also had high area deprivation (ie, poor housing conditions, health outcomes, and socioeconomic disadvantage). These findings remained significant after controlling for individual-level characteristics such as age, gender, race, ethnicity, education level, and household income.

Several social determinant frameworks have linked exposure from neighborhood-level factors, to the activation of mechanisms that contribute to chronic stressors, which ultimately lead to increased mental health symptoms and psychosis risk.^5,27^ Future studies should explore potential mechanisms similar to those outlined in the social determinants model for psychosis such as lack of safety, high attentional demands, social capital, and limited environmental enrichment to shed light on potential clinical targets that serve to interrupt the adverse impact of neighborhoods. While it is important for clinicians to capture contextual factors and understand how this information can inform treatment, it is simply not enough to make adaptations to treatment (eg, CSC) without addressing multiple intersecting determinants that extend beyond individual-level risk factors. To create sustainable change, these multidimensional problems will require systemic, multifaceted solutions to tackle detrimental neighborhood conditions through comprehensive approaches. Policy changes must address housing conditions and lack of infrastructure (eg, resources, economic growth) that contribute to social disadvantage. For instance, removing structural barriers to accessing public transportation and the provision of subsidized public transit passes, especially for those who reside in low socioeconomic communities, has the potential to increase mobility, access to services, and improve economic opportunity.^36^

### Limitations

Several limitations should be considered. Although using composite indicators is advantageous for capturing unique multifaceted latent constructs (eg, social and economic determinants) while addressing statistical concerns related to model over specification, the lack of standardized operationalization across studies and their agnosticism of the extent to which individual indictors contribute to overall model fit is a limitation. A cross-sectional design was used, whereby mental health outcome measures and current address data were collected from individual participants at a single time point. Indicators used to derive profiles were drawn from data spanning the broader recruitment time frame (2017 to 2021). Because residential histories were not collected, the alignment between an individual’s neighborhood exposure and mental health outcomes may not be precise. Future longitudinal analyses with repeated neighborhood and individual measurements could enable stronger claims about the temporality of observed associations. The present study represents the unique geographical composition of Washington State; given this, neighborhood profiles may not ideally map onto those observed in other states.

## Conclusions

This study suggests that individuals with FEP residing in urban neighborhoods that are characterized as socially and economically disadvantaged and exposed to high levels of environmental pollutants present with more severe symptoms than those residing in rural and less disadvantaged urban areas. These findings underscore the need to explore how the cumulative effect of neighborhood exposomes is associated with psychosis onset, outcomes, and subsequent mental health symptoms. The clinical implications call for a greater focus on contextual factors and strategies within CSC and implementation of strategies that build resilience against detrimental neighborhood effects.

## Notes

Supplement 1.
eMethods.
**eTable 1.** Indicators Included in Washington Tracking Network’s Information by Location Economic Determinants Composite Metric **eTable 2.** Indicators Included in Washington Tracking Network’s Information by Location Social Determinants Composite Metric **eTable 3.** Indicators Included in Washington Tracking Network’s Information by Location Poor Health Outcomes Composite Metric **eTable 4.** Indicators Included in Washington Tracking Network’s Information by Location Housing Conditions Composite Metric **eTable 5.** Indicators Included in Washington Tracking Network’s Information by Location Housing Conditions Composite Metric **eTable 6.** Class Solutions and Model Fit Indices for Latent Profile Analysis of Washington Neighborhoods **eFigure.** Class Solutions and Model Fit Indices for Latent Profile Analysis of Washington Neighborhoods
eReferences.

Supplement 2.
Data Sharing Statement
