# Supplementary material for: Multidimensional Approach to Exploring Neighborhood Determinants and Symptom Severity Among Individuals With Psychosis
Source: JAMA Netw Open. 2024 May 15;7(5):e2410269. doi: 10.1001/jamanetworkopen.2024.10269 (PMC11096989; doi:10.1001/jamanetworkopen.2024.10269)
Supplement: Supplement 1. — eMethods. eTable 1. Indicators Included in Washington Tracking Network’s Information by Location Economic Determinants Composite Metric eTable 2. Indicators Included in Washington Tracking Network’s Information by Location Social Determinants Composite Metric eTable 3. Indicators Included in Washington Tracking Network’s Information by Location Poor Health Outcomes Composite Metric eTable 4. Indicators Included in Washington Tracking Network’s Information by Location Housing Conditions Composite Metric eTable 5. Indicators Included in Washington Tracking Network’s Information by Location Housing Conditions Composite Metric eTable 6. Class Solutions and Model Fit Indices for Latent Profile Analysis of Washington Neighborhoods eFigure. Class Solutions and Model Fit Indices for Latent Profile Analysis of Washington Neighborhoods eReferences. [file jamanetwopen-e2410269-s001.pdf]

Supplemental Online Content

Oluwoye O, Puzia M, Lissau A, Amram O, Weeks DL. Multidimensional approach to exploring neighborhood determinants and symptom severity among individuals with psychosis. *JAMA Netw Open.* 2024;7(5):e2410269.
doi:10.1001/jamanetworkopen.2024.10269

eMethods.

eTable 1. Indicators Included in Washington Tracking Network's Information by Location Economic Determinants Composite Metric

eTable 2. Indicators Included in Washington Tracking Network's Information by Location Social Determinants Composite Metric

eTable 3. Indicators Included in Washington Tracking Network's Information by Location Poor Health Outcomes Composite Metric

eTable 4. Indicators Included in Washington Tracking Network's Information by Location Housing Conditions Composite Metric

eTable 5. Indicators Included in Washington Tracking Network's Information by Location Housing Conditions Composite Metric

eTable 6. Class Solutions and Model Fit Indices for Latent Profile Analysis of Washington Neighborhoods

eFigure. Class Solutions and Model Fit Indices for Latent Profile Analysis of Washington Neighborhoods

eReferences.

This supplemental material has been provided by the authors to give readers additional information about their work.

© 2024 Oluwoye O et al. *JAMA Network Open.*

eMethods.

LPA Indicators from the Washington Tracking Network Data Portal

Washington State Department of Health's Washington Tracking Network (WTN) is a state-level data system supported by the US Centers for Disease Control and Prevention's National Environmental Public Health Tracking Program that includes an extensive collection of public health datasets from various sources including environmental monitoring, hospital records, census surveys, and GIS mapping (WS-DOT, 2019). Data within the WTN are compiled from a variety of sources, including environmental monitoring stations, GIS locators, hospital admissions, and the US census and American Community Survey. In the current study, we queried data for three neighborhood-related indicators: healthcare shortage areas, access to outdoor recreation space, and poor land use mix. For each indicator, the raw data values are ranked by census tract. Each census tract is assigned a percentile based on the ranking of the indicator. To facilitate comparability with other composite variables (see LPA Indicators from the Washington Tracking Network's Information by Location tool, below) percentiles were transformed into decline rankings for analyses. For all indicators, additional details are available within the WTN data portal.

Access to Outdoor Recreation Space

Park location data was derived from ESRI and NAVTEQ sources and represent local parks, county parks regional parks, state parks and forest lands, beaches with public access, national parks, forests and wildlife refuges in 2015. The Washington State Office of Financial Management (OFM) uses mathematical models of births, deaths, and migration to make forecasts based on numbers obtained from the US Census Bureau. A one-mile buffer around all parks was created, and the percentage area of each census block that is within a park buffer was calculated. The corresponding percentage of the population younger than 18 years old within a park buffer was calculated by taking the total population younger than 18 divided by the total population for all census blocks that make up a census tract.

Poor Land-Use Mix

Land use mix is calculated based on data from Washington State Department of Ecology's 2010 land use geodatabase. The land use mix score considers educational uses, entertainment uses, single-family residence uses, multi-family residence uses, retail uses, and office uses. The land use mix was calculated using an adaptation of a formula published by

© 2024 Oluwoye O et al. *JAMA Network Open.*

Frank et. al. 2006 as a component of a walkability score: The measure displays a score from zero to 100 with zero being only one land use type and 100 being an equal distribution of six land use types (education, entertainment, single-family residence, multi-family residence, retail, office). Within the WTN, decile rankings reflect "Poor Land-Use Mix," such that higher rankings correspond to lower-diversity land use.

Healthcare Professional Shortage Areas

Health Provider Shortage were determined based on data on Primary Care, Mental Health, and Dental Healthcare Shortage Area (HPSA) datasets maintained by the Health Resources and Services Administration's (HRSA) Division of Policy and Shortage Designation. Scores from primary health care, mental health, and dental health HPSAs were summed across census tracts. Scores for each shortage type range from 0 to 26. The scores are based on the number of providers per capita and information on the intensity of the need for providers in that area.

LPA Indicators from the Washington Tracking Network's Information by Location tool

In addition to raw data and percentile scores on individual data indicators, the WTN has developed composite metrics for the Map (University of Washington Department of Environmental & Occupational Health Sciences, 2019), which are available via the WTN's Information by Location (IBL) mapping tool. To create composites, the WTN percentile scores for all indicators are averaged within a given tract and averages are ranked using deciles (1 decile = 10 percent). Each decile represents about 10 percent of the values in the dataset. In the current study, we used the IBL to collect information on economic characteristics, social determinants of health, nearby housing conditions and land use, access to transportation, area environmental exposure, and population health outcomes. Additional details about indicators for each are available within the WTN data portal.

Economic Determinants Composite

Data informing the Economic Determinants composite variable were drawn from the U.S. Census Bureau's American Community Survey 5-year estimates for 2015-19 (U.S. Census Bureau, 2019). Indicators included in the composite metric are shown in eTable 1.

Social Determinants Composite

© 2024 Oluwoye O et al. *JAMA Network Open.*

Data informing the Social Determinants variable were drawn from the U.S. Census Bureau's American Community Survey 5-year estimates for 2015-19 (U.S. Census Bureau, 2019). Indicators included in the composite metric are shown in eTable 2.

Poor Health Outcomes Composite

Data informing Poor Health Outcomes were drawn from Washington State Department of Health (WA DOH). Indicators included in the composite metric are shown in eTable 3.

Housing Conditions Composite

Data informing the Housing Conditions variable were drawn from the U.S. Census Bureau's American Community Survey 5-year estimates for 2015-19 (U.S. Census Bureau, 2019). Indicators included in the composite metric are shown in eTable 4.

Environmental Exposures Composite

Data informing the Environmental Exposures composite was drawn from the Washington State Department of Ecology's 2014 Comprehensive Emissions Inventory (Washington State Department of Ecology, 2022) and Washington State University's Air Indicator Report for Public Awareness and Community Tracking (AIRPACT) modeling domain (Vaughn et al, 2002), Washington State Department of Transportation's vehicular traffic data monitoring program (WA DOT, 22019), and the Environmental Protection Agency's Risk Screening tool (U.S. EPA, 2021). Indicators included in the composite metric are shown in eTable 5.

LPA Indicators from Other Sources

Rural Urban Commuting Area Codes

Rurality was determined using the Rural-Urban Commuting Area (RUCA) taxonomy, a classification system developed by the United States Department of Agriculture (USDA, 2013) that categorizes U.S. census tracts based on their degree of urbanization and commuting patterns (USDA, 2013). RUCA codes are based on measures of population density, urbanization, and commuter flows. These codes range from 1 (most urban) to 10 (most rural). Categorically, codes between 1 and 3 reflect metropolitan areas, between 4 and 6 micropolitan areas, between 7 and 9 small-town areas, and codes of 10 reflect rural areas.

Food Access Research Atlas

The Food Access Research Atlas was developed by the USDA to provide detailed information on food access across the U.S. and highlight areas where individuals may have

© 2024 Oluwoye O et al. *JAMA Network Open.*

difficulty accessing healthy and affordable food options (USDA, 2019). In the current study, tract access was measured as the percentage of the population living further than one mile away from a supermarket.

National Walkability Index

The National Walkability Index is a quantitative assessment tool used to evaluate and rank the pedestrian-friendliness of different neighborhoods (U.S. EPA, 2017). It considers sidewalk availability, connectivity, traffic safety, proximity to amenities, public transportation access, street design, safety from crime, and walkability infrastructure investments.

Department of Transportation, Transportation Barriers Score

The degree of transportation barriers for each census tract was characterized using scores developed by the Washington State Department of Transportation (DOT; U.S. DOT, 2022). The DOT transportation barriers scores quantify the difficulty of travel to essential services based on road metrics, transit availability, and travel time to key destinations like grocery stores, doctors, and schools. Census tracts receive a score from 0 to 100, with lower scores indicating more barriers to transportation such as limited access to vehicles, long travel times, and lack of public transit options. The scores were developed using travel demand modeling and accessibility analysis based on the street network, transit network, and locations of essential services.

© 2024 Oluwoye O et al. *JAMA Network Open.*

eTable 1. *Indicators included in Washington Tracking Network's Information by Location Economic Determinants Composite Metric*

Indicator	Description	Source
Children Living in Poverty	This measure represents the percent of children under 18 years old who have experienced poverty within the past 12 months (REF. The Census Bureau uses a set of money income thresholds that vary by family size and composition to determine who is in poverty. If a family's total income is less than the family's threshold, then that family and every individual in it is considered in poverty.	Washington Tracking Network's Information by Location mapping tool<br>U.S. Census Bureau's American Community Survey 5 year estimates 2015-19: S1701 - Poverty Status in the Past 12 Months<br>How the Census Bureau Measures Poverty
Population Living 185% Below Poverty	This indicator uses data on the percent of the population living below 185 percent of the federal poverty level. The Census Bureau uses a set of money income thresholds that vary by family size and composition to determine who is in poverty. If a family's total income is less than the family's threshold, then that family and every individual in it is considered in poverty.	Washington Tracking Network's Information by Location mapping tool<br>U.S. Census Bureau's American Community Survey 5 year estimates 2015-19: S1701 - Poverty Status in the Past 12 Months<br>How the Census Bureau Measures Poverty
Population 19 to 64 with No Health Insurance	This measure represents the total civilian non-institutionalized population, ages 19 to 64 that do not have health insurance. Health insurance includes both private and public (e.g. Medicaid).	Washington Tracking Network's Information by Location mapping tool<br>U.S. Census Bureau's American Community Survey 5 year estimates 2015-19: S2701 -

© 2024 Oluwoye O et al. *JAMA Network Open.*

		Selected Characteristics of Health Insurance Coverage
Single Parent Households	This measure displays the percentage of households with children under 18 years of age with a single parent or guardian.	Washington Tracking Network's Information by Location mapping tool<br>U.S. Census Bureau's American Community Survey 5 year estimates 2015-19: DP02 - Selected Social Characteristics
Unaffordable Housing	This measure represents the percentage householders spend on housing costs. There are three categories under "Selected Monthly Costs as Percentage of Household Income": households with mortgages, households without mortgages, and rentals. "Unaffordable housing" is defined as households spending greater than 30 percent of their income on housing costs. The housing burden indicator displays the modeled percent of income spent on housing for a four-person household making the median household income.	Washington Tracking Network's Information by Location mapping tool<br>U.S. Census Bureau's American Community Survey 5 year estimates 2015-19: DP04 - Selected Housing Characteristics
Population Unemployed	This indicator uses the percent of the population over the age of 16 that is unemployed and eligible for the labor force. This indicator excludes retirees, students, homemakers, institutionalized persons except for prisoners, those not looking for work, and	Washington Tracking Network's Information by Location mapping tool<br>U.S. Census Bureau's American Community Survey 5 year estimates 2015-19: DP03 - Selected Economic Characteristics

	military personnel on active duty.	

© 2024 Oluwoye O et al. *JAMA Network Open.*

eTable 2. *Indicators included in Washington Tracking Network's Information by Location Social Determinants Composite Metric*

Indicator	Description	Source
Limited English Proficiency	This measure displays the percentage of the population five years and older that speak English less than "very well" and "not at all" (LEP).	Washington Tracking Network's Information by Location mapping tool U.S. Census Bureau's American Community Survey 5 year estimates 2015-19: B16004 - Age by Language Spoken at Home by Ability to Speak English
No Access to a Private Vehicle	This measure displays the total number and percentage of households with no access to a vehicle.	Washington Tracking Network's Information by Location mapping tool U.S. Census Bureau's American Community Survey 5 year estimates 2015-19: DP04 - Selected Housing Characteristics
No High School Diploma	This measure displays the percent of people who have not received a high school diploma or GED by the age of 25.	Washington Tracking Network's Information by Location mapping tool U.S. Census Bureau's American Community Survey 5 year estimates 2015-19: DP02 - Selected Social Characteristics
Population 65+ Living Alone	This measure displays the population aged 65 and older living alone as a total number and as a percentage of the total population.	Washington Tracking Network's Information by Location mapping tool U.S. Census Bureau's American Community Survey 5 year estimates 2015-19: DP02 - Selected Social Characteristics
Population with a Disability	This measure displays the percentage of the population	Washington Tracking Network's Information by Location

© 2024 Oluwoye O et al. *JAMA Network Open.*

	with a disability (i.e., hearing difficulty, vision difficulty, cognitive difficulty, ambulatory difficulty, self-care difficulty, or independent-living difficulty)	mapping tool<br>U.S. Census Bureau's American Community Survey 5 year estimates 2015-19: DP02 - Selected Social Characteristics<br>Disability - Census Reporter

© 2024 Oluwoye O et al. *JAMA Network Open.*

eTable 3. *Indicators included in Washington Tracking Network's Information by Location Poor Health Outcomes Composite Metric*

Indicator	Description	Source
Cancer Deaths – all	This measure was developed using cardiovascular disease mortality data collected from death certificates of state residents, including the deaths of Washington state residents that died in other states or in Canada. This measure reflects age-adjusted cancer mortality rates per 100,000. It includes deaths attributable to all cancer types, combined. The causes of death are based on the underlying cause of death as determined by the, physician, physician assistant, advanced registered nurse practitioner, or medical examiner.	Washington Tracking Network's Information by Location mapping tool<br>Washington State Department of Health, Center for Health Statistics: Death Certificate Data
Death from Cardiovascular Disease	This measure was developed using cardiovascular disease mortality data collected from death certificates of state residents, including the deaths of Washington state residents that died in other states or in Canada. Mortality from cardiovascular diseases (NCHS 113: Major cardiovascular diseases; see Miniño & Klein, 2010) represents the proportion of deaths to a population due to	Washington Tracking Network's Information by Location mapping tool<br>Washington State Department of Health, Center for Health Statistics: Death Certificate Data

© 2024 Oluwoye O et al. *JAMA Network Open.*

	cardiovascular disease. The rate represents the age adjusted rate per 100,000 population. The causes of death are based on the underlying cause of death as determined by the, physician, physician assistant, advanced registered nurse practitioner, or medical examiner.	
Low Birth Weight – Combined	This measure was developed using birth and fetal death data compiled from information on birth and fetal death certificates. This index reflects the number of live born singleton (one baby) infants born at term (at or above 37 completed weeks of gestation) with a birth weight of less than 2500 grams (about 5.5 pounds). The low birth weight (LBW) rate (percentage) is the count divided by the total number of live born singleton infants born at term to resident mothers.	Washington Tracking Network's Information by Location mapping tool<br>Washington State Department of Health, Center for Health Statistics: Community Health Assessment Tool (CHAT)
Lower Life Expectancy at Birth	This measure was developed using all-cause mortality data, collected from death certificates of state residents, including the deaths of Washington state residents that died in other states or in Canada. Life expectancy is defined as the number of years a newborn can expect to live if the current age-specific death rates	Washington Tracking Network's Information by Location mapping tool<br>Washington State Department of Health, Center for Health Statistics: Death Certificate Data

© 2024 Oluwoye O et al. *JAMA Network Open.*

	remain constant. Life expectancy calculations of older age groups show the result as the number of years of additional life a person in that age group can expect to live if the current death rates for that age group remain constant.	
Premature Death	This measure was developed using all-cause mortality data, collected from death certificates of state residents, including the deaths of Washington state residents that died in other states or in Canada. Premature mortality uses the age when a person died based on a life expectancy to age 65 and takes those years as the number of years of potential life lost (YPLL). This measure represents the YPLL relative to age 65. Rates are calculated per 100,000 population.	Washington Tracking Network's Information by Location mapping tool<br>Washington State Department of Health, Center for Health Statistics: Community Health Assessment Tool (CHAT)

© 2024 Oluwoye O et al. *JAMA Network Open.*

eTable 4. *Indicators included in Washington Tracking Network's Information by Location Housing Conditions Composite Metric*

Indicator	Description	Source
Lead Risk from Housing	This indicator models potential lead exposure. It reflects the number and percent of housing units built before 1980, including single homes and multiple residence units such as apartments. The age of a building by itself does not reflect the actual exposure to lead. The age of a home is a marker of risk for the presence of lead paint because paint typically contained high levels of lead in the decades leading up to 1980. Each era of housing is adjusted by a factor that reflects proportionate risk for that era (housing built: before 1940 = 0.68; 1940-1959= 0.43; 1960-1979= 0.08; see Jacobs, 2002).	Washington Tracking Network's Information by Location mapping tool<br>U.S. Census Bureau's American Community Survey 5 year estimates 2015-19: DP04 - Selected Housing Characteristics
Mobile Homes	This measure reflects the percentage of the owner- or renter-occupied housing units that are mobile homes.	Washington Tracking Network's Information by Location mapping tool<br>U.S. Census Bureau's American Community Survey 5 year estimates 2015-19: DP04 - Selected Housing Characteristics
Overcrowded Housing	This measure reflects the percentage of people living in housing where there is more than one person per room.	Washington Tracking Network's Information by Location mapping tool<br>U.S. Census Bureau's American

© 2024 Oluwoye O et al. *JAMA Network Open.*

		Community Survey 5 year estimates 2015-19: DP04 - Selected Housing Characteristics
Unaffordable Housing	This measure represents the percentage householders spend on housing costs. There are three categories under "Selected Monthly Costs as Percentage of Household Income": households with mortgages, households without mortgages, and rentals. “Unaffordable housing” is defined as households spending greater than 30 percent of their income on housing costs. The housing burden indicator displays the modeled percent of income spent on housing for a four-person household making the median household income.	Washington Tracking Network’s Information by Location mapping tool<br>U.S. Census Bureau’s American Community Survey 5 year estimates 2015-19: DP04 - Selected Housing Characteristics
Unoccupied Housing	This measure displays the number and percentage of unoccupied housing units.	Washington Tracking Network’s Information by Location mapping tool<br>U.S. Census Bureau’s American Community Survey 5 year estimates 2015-19: DP04 - Selected Housing Characteristics

© 2024 Oluwoye O et al. *JAMA Network Open.*

eTable 5. *Indicators included in Washington Tracking Network's Information by Location Housing Conditions Composite Metric*

Indicator	Description	Source
Diesel Exhaust PM2.5 Emissions	Estimates of all diesel exhaust $PM_{2.5}$ emissions (annual tons) were mapped to the AIRPACT modeling domain, which uses 4km x 4km grid cells. Major point source emissions were directly allocated to the grid cell in which they are located. Other emission sources (e.g. non-point and mobile) were allocated to grid cells based on spatial surrogates developed for AIRPACT. Each census tract was assigned the maximum emissions estimate of any grid cells that intersect it.	Washington Tracking Network's Information by Location mapping tool<br>Washington State Department of Ecology's 2014 Comprehensive Emissions Inventory: Field Diesel $PM_{2.5}$ annual tons 2014 estimates<br>AIRPACT modeling domain
Ozone Concentration	This measure uses 8-hour ozone design values interpolated at 4km x 4km grid cells from July 2014-June 2017. The form of the ozone design value is the annual fourth-highest daily maximum 8-hour concentration (D8M), averaged over three years. Design values were interpolated using the relationship between ozone design values measured at air quality agency monitoring sites and median forecast D8M ozone from the AIRPACT forecast model. Ozone design values were	Washington Tracking Network's Information by Location mapping tool<br>NW-AIRQUEST Regional Background Design Values, 2014-2017 estimates<br>AIRPACT modeling domain

© 2024 Oluwoye O et al. *JAMA Network Open.*

	interpolated across Washington with Empirical Bayesian Kriging Regression Prediction using measured design values as the dependent variable and median forecast D8M as the explanatory variable. Each census tract was assigned the interpolated ozone design value of the most populated grid cell that intersects that tract.	
PM2.5 Concentration	This measure uses the mean and 98th percentile daily $PM_{2.5}$ concentrations estimated at 4km x 4km grid cells from July 2014-June 2017. The 3-year mean and 3-year 98th percentile daily $PM_{2.5}$ concentrations are surrogates for the annual and 24-hour $PM_{2.5}$ design values, respectively. Mean and 98th percentile concentrations were interpolated at grid cells using the relationship between mean/98th percentile $PM_{2.5}$ measured at air quality agency monitoring sites and median daily forecast $PM_{2.5}$ from the AIRPACT forecast model. The monitor/model ratio at each monitoring site was calculated and then interpolated across Washington using Empirical Bayesian Kriging. The	Washington Tracking Network's Information by Location mapping tool<br>Washington State Department of Ecology: Field PM2.5 2014 – 2017 estimates

	interpolated ratios were multiplied by median daily forecast $PM_{2.5}$ from AIRPACT at each 4km x 4km grid cell to yield interpolated mean and 98th percentile $PM_{2.5}$. Each census tract was assigned the maximum interpolated mean and 98th percentile $PM_{2.5}$ value of any grid cells that intersect it. Mean and 98th percentile values at census tracts were each normalized to a scale of [0-1] and summed to give each census tract a single $PM_{2.5}$ score.	
Proximity to Heavy Traffic Roadways	This measure reflects the maximum distance-weighted traffic along Washington highways for each census tract. Average Annual Daily Traffic (AADT) on highways is from a network of permanent and short-duration traffic counters. The units are maximum highway AADT (vehicles per day) / Distance to highway (km) or vehicles/day/km. Census tract files were mapped onto spatial files of 2019 roadway traffic sections data from the WSDOT map center.	Washington Tracking Network's Information by Location mapping tool<br>Washington Geospatial Open Data Portal: WSDOT - Historic Traffic Sections 2019
Toxic Releases from Facilities (RSEI Model)	This indicator shows the toxicity-weighted concentrations of chemical releases to air from	Washington Tracking Network's Information by Location mapping tool

© 2024 Oluwoye O et al. *JAMA Network Open.*

	facility emissions and off-site incineration. Data was downloaded from Risk Screening Environmental Indicators (RSEI) where air releases are modeled by the Toxics Release Inventory (TRI) program. The Geographic Microdata is a model of Air pollution releases that are plotted on 810-meter grid cells aggregated at the census tract level for 2018, 2019, and 2020. RSEI models the toxicity-weighted concentration into air from TRI sites for census tracts. Three-year averages were calculated for each tract.	U.S. Environmental Protection Agency's Environmental Justice Screening and Mapping Tool RSEI Geographic Microdata (RSEI-GM) | US EPA

© 2024 Oluwoye O et al. *JAMA Network Open.*

eTable 6. *Class Solutions and Model Fit Indices for Latent Profile Analysis of Washington Neighborhoods*

Classes	AIC	AWE	BIC	CLC	KIC	BLRT *p*
1	49,687.49	50,059.16	49,814.33	49,641.49	49,714.49	--
2	45,901.04	46,475.30	46,096.58	45,828.86	45,941.04	.01
3	42,376.19	43,152.82	42,640.43	42,278.05	42,429.19	.01
4	40,719.74	41,698.76	41,052.69	40,595.61	40,785.74	.01
5	39,406.32	40,587.76	39,807.97	39,256.18	39,485.32	.01
6	38,272.20	39,656.03	38,742.55	38,096.07	38,364.20	.01
7	37,769.58	39,355.84	38,308.63	37,567.42	37,874.58	.01
8	37,801.77	39,590.46	38,409.52	37,573.59	37,919.77	> .99

Notes. AIC = Akaike Information Criterion; Approximate Weight of Evidence Criterion; BIC = Bayesian Information Criterion; BLRT = bootstrapped likelihood ratio test; CLC = Classification Likelihood Criterion; Kullback Information Criterion.

© 2024 Oluwoye O et al. *JAMA Network Open.*

eFigure. *Class Solutions and Model Fit Indices for Latent Profile Analysis of Washington Neighborhoods*

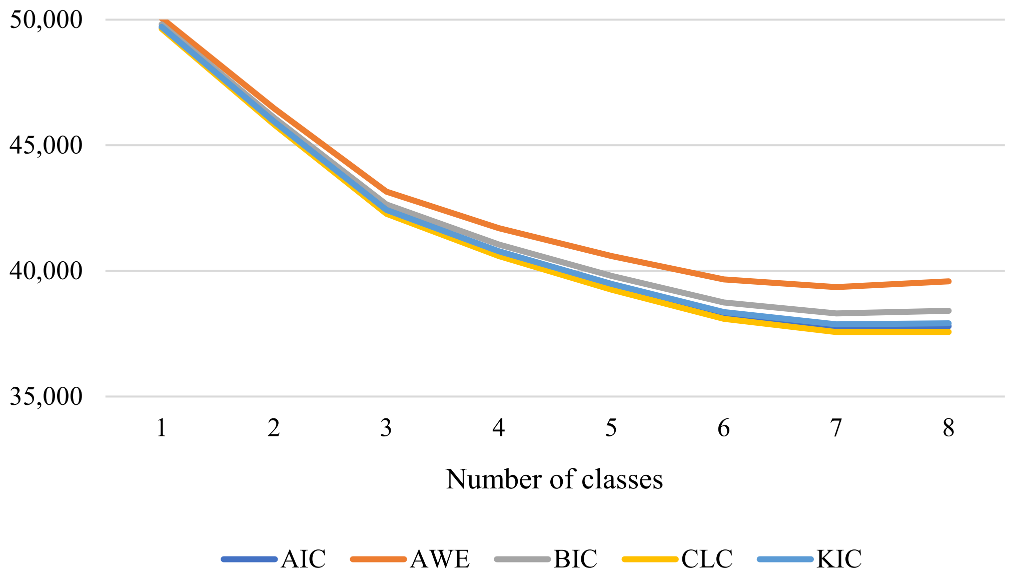

Notes. AIC = Akaike Information Criterion; Approximate Weight of Evidence Criterion; BIC = Bayesian Information Criterion; CLC = Classification Likelihood Criterion; Kullback Information Criterion.

© 2024 Oluwoye O et al. *JAMA Network Open.*

eReferences

Frank, L. D., Sallis, J. F., Conway, T. L., Chapman, J. E., Saelens, B. E., & Bachman, W. (2006). Many pathways from land use to health: Associations between neighborhood walkability and active transportation, body mass index, and air quality. Journal of the American planning Association, 72(1), 75-87.

Jacobs, D. E., Clickner, R. P., Zhou, J. Y., Viet, S. M., Marker, D. A., Rogers, J. W., ... & Friedman, W. (2002). The prevalence of lead-based paint hazards in US housing. *Environmental health perspectives*, *110*(10), A599-A606.

Miniño AM, Klein RJ. Health mortality from major cardiovascular diseases: United States, 2007. Health E-Stats. National Center for Health Statistics. March 2010.

University of Washington Department of Environmental & Occupational Health Sciences. Washington Environmental Health Disparities Map: technical report. Seattle; 2019.

U.S. Census Bureau (2019). Detailed Tables. *2019 American Community Survey 5-year estimates data profiles*. Retrieved from https://data.census.gov/all?d=ACS+5-Year+Estimates+Detailed+Tables

U.S. Department of Agriculture. (2013). Rural-Urban Commuting Area codes (RUCA). Retrieved from https://www.ers.usda.gov/data-products/rural-urban-commuting-area-codes

U.S. Department of Agriculture, Economic Research Service. (2019). Food Access Research Atlas. Retrieved from https://www.ers.usda.gov/data-products/food-access-research-atlas

U.S. Environmental Protection Agency (EPA), 2021. EJScreen Technical Documentation.

Vaughan, J., Lamb, B., Wilson, R., Bowman, C., Figueroa-Kaminsky, C., Otterson, S., ... & Albright, M. (2002). 4.1 AIRPACT: A real-time air quality forecast system for the pacific northwest.

Washington Department of Transportation (DOT), 2021. Traffic Data Reporting System.

Washington State Department of Ecology, 2022. Washington State Greenhouse Gas Emissions Inventory: 1990–2019. https://apps.ecology.wa.gov/publications/summarypages/2202054.html

Washington State Department of Health (WS-DOH), 2019. Washington Tracking Network (WTN): A complete list of all data on WTN's query tool.

© 2024 Oluwoye O et al. *JAMA Network Open.*

https://doh.wa.gov/sites/default/files/legacy/Documents/4000/334-411-WTNAllMeasures.pdf

© 2024 Oluwoye O et al. *JAMA Network Open.*
