# Supplementary material for: Multidimensional Approach to Exploring Neighborhood Determinants and Symptom Severity Among Individuals With Psychosis
Source: JAMA Netw Open. 2024 May 15;7(5):e2410269. doi: 10.1001/jamanetworkopen.2024.10269 (PMC11096989; doi:10.1001/jamanetworkopen.2024.10269)
Supplement: Supplement 2. — Data Sharing Statement [file jamanetwopen-e2410269-s002.pdf]

Data Sharing Statement

Oluwoye. Multidimensional Approach to Exploring Neighborhood Determinants and Symptom Severity Among Individuals With Psychosis. *JAMA Netw Open*. Published May 15, 2024. doi:10.1001/jamanetworkopen.2024.10269

Data
Data available: No
